# Analytical parameters and validation of homopolymer detection in a pyrosequencing-based next generation sequencing system

**DOI:** 10.1186/s12864-018-4544-x

**Published:** 2018-02-21

**Authors:** Gergely Ivády, László Madar, Erika Dzsudzsák, Katalin Koczok, János Kappelmayer, Veronika Krulisova, Milan Macek, Attila Horváth, István Balogh

**Affiliations:** 1 Department of Laboratory Medicine, University of Debrecen, Nagyerdei krt. 98, Debrecen, H-4032 Hungary; 2 Department of Biology and Medical Genetics, Second Faculty of Medicine and University Hospital Motol, Charles University, Prague, Czech Republic; 3 Genomic Medicine and Bioinformatic Core Facility, University of Debrecen, Debrecen, Hungary; 4 Division of Clinical Genetics, University of Debrecen, Nagyerdei krt. 98, Debrecen, H-4032 Hungary

**Keywords:** Pyrosequencing, Homopolymer detection, Cystic fibrosis

## Abstract

**Background:**

Current technologies in next-generation sequencing are offering high throughput reads at low costs, but still suffer from various sequencing errors. Although pyro- and ion semiconductor sequencing both have the advantage of delivering long and high quality reads, problems might occur when sequencing homopolymer-containing regions, since the repeating identical bases are going to incorporate during the same synthesis cycle, which leads to uncertainty in base calling. The aim of this study was to evaluate the analytical performance of a pyrosequencing-based next-generation sequencing system in detecting homopolymer sequences using homopolymer-preintegrated plasmid constructs and human DNA samples originating from patients with cystic fibrosis.

**Results:**

In the plasmid system average correct genotyping was 95.8% in 4-mers, 87.4% in 5-mers and 72.1% in 6-mers. Despite the experienced low genotyping accuracy in 5- and 6-mers, it was possible to generate amplicons with more than a 90% adequate detection rate in every homopolymer tract. When homopolymers in the *CFTR* gene were sequenced average accuracy was 89.3%, but varied in a wide range (52.2 – 99.1%). In all but one case, an optimal amplicon-sequencing primer combination could be identified. In that single case (7A tract in exon 14 (c.2046_2052)), none of the tested primer sets produced the required analytical performance.

**Conclusions:**

Our results show that pyrosequencing is the most reliable in case of 4-mers and as homopolymer length gradually increases, accuracy deteriorates. With careful primer selection, the NGS system was able to correctly genotype all but one of the homopolymers in the *CFTR* gene. In conclusion, we configured a plasmid test system that can be used to assess genotyping accuracy of NGS devices and developed an accurate NGS assay for the molecular diagnosis of CF using self-designed primers for amplification and sequencing.

**Electronic supplementary material:**

The online version of this article (10.1186/s12864-018-4544-x) contains supplementary material, which is available to authorized users.

## Background

In genomics, a homopolymer (HP) is a sequence of consecutive identical bases. Approximately 1.43 million HPs (also known as mononucleotide microsatellites) exist in the human exome, with the size of 4-mer and up. This also means that an average of eight such HP sequences can be found in every exon. They are believed to play roles in transcriptional regulation and recombination [1, 2], and the vast majority (96.7%) of them are in the range of 4-mer to 6-mer. HP sequences composed of A:T base pairs are over-represented in the human genome compared to G:C HPs [3–5]. Although both pairs show structural stability [6, 7], these loci in the genome are highly mutagenic and have been characterized as hotspots for length change mutations [8–10], which has, presumably, contributed to their reduced occurrence in the exome over time.

Certain sequencing-by-synthesis based next generation sequencing (NGS) techniques have a relatively high error rate in determining homopolymer regions, due to the principles used for detection. In pyrosequencing [11–13] or ion semiconductor sequencing [14], the signal which depends on the emitted light or the concentration of released hydrogen ions, respectively, should be directly proportional to the number of incorporated bases during a single dNTP dispensation. However, since linearity starts to diminish in HP stretches exceeding four bases, erroneous over- and under calls are going to happen [15–17]. Since HPs are more prone to insertion and deletion mutations (indels), problems are going to aggravate, when utilizing pyrosequencers or ion semiconductor chemistry in diagnostic procedures [18].

There are numerous bioinformatic correction tools to separate artifacts from true genetic variations. Some of these algorithms are based on clustering the flowgrams; for example Denoiser, which utilizes rank-abundance distributions, or PyroNoise/AmpliconNoise, which calculates a likelihood using empirically derived error distributions [19]. Acacia’s main focus is on HP sequences and the algorithm uses a dynamically updated cluster consensus when aligning reads [20]. Coral and ECHO are multiple alignment based techniques [21], while HECTOR is a homopolymer spectrum based error corrector, with a multistage correction workflow [22]. Another useful software is FlowClus, which provides feedback on the denoising process, allowing the user to apply more suitable analysis parameters for the particular dataset [23]. The most recent tools, such as NoDe (Noise Detector) and DUDE-Seq are believed to produce even lower error rates and are more time-efficient [24, 25]. Even if sophisticated correction tools [22, 26, 27] are used to overcome the difficulties of detection and significantly improve accuracy, it is still very important to estimate the capability of the corresponding NGS system to correctly determine HPs. To avoid uncertainty in the diagnostic testing of patient samples, it is also recommended that the maximum length of stable HP detection, for reasonable identification of indel mutations in such sequences, be defined before using the NGS instrument in routine clinical practice [28].

The coding region of the cystic fibrosis transmembrane regulator gene (*CFTR)* contains 24 homopolymer stretches, involving 17 out of 27 exons. In conjunction with the whole genome, T and A homopolymers vastly outnumber G and C homopolymers; 14 thymine, 8 adenine, 2 guanine and no cytosine HPs are present. In exon 14 there is a seven adenine long homopolymer region (c.2046_2052) and genetic alterations affecting this region could create poly-A tracts of different sizes, e.g. the pathogenic mutation c.2051_2052delAAinsG (2183delAAinsG) results in a five, while c.2052delA (2184delA) results in a six adenine long homopolymer segment. In case of the relatively frequent c.2052_2053insA (2184insA) mutation an eight adenine long homopolymer stretch is formed.

To assess the analytical validity of pyrosequencing-based NGS technology and to test the optimization possibilities, two experimental test systems were developed. In the first, homopolymer sessions were introduced into plasmid vectors and the resultant plasmids were used as templates for subsequent sequencing experiments. Next we analyzed the *CFTR* gene to also assess the analytical performance of a newly developed pyrosequencing-based assay on patient samples. In this second experiment, all homopolymer-containing and non-HP *CFTR* exons were tested. These samples originated from cystic fibrosis (CF) patients with known *CFTR* mutation status. Assessment of the Ion Torrent platform was carried out in a similar way and the results were recently published [29].

Another reason for investigating the *CFTR* gene was that the above mentioned CF producing mutation (2184insA) is very common in certain geographical regions of Europe [30], including Hungary [31, 32]. Despite, current CF mutation detection kits do not cover this particular mutation site.

## Methods

**Fig. 1:**
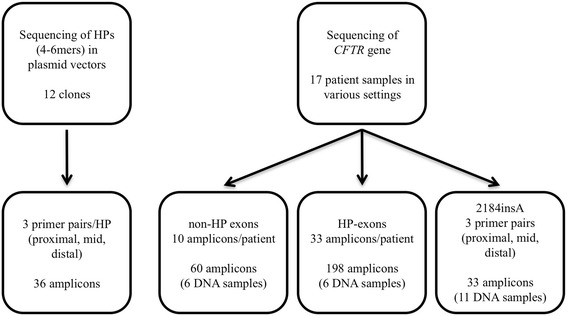
Experimental setup. In the plasmid system 4-to 6-mer homopolymers (HPs) were generated and tested using different primer sets. In the CFTR assay, 17 samples with known genotype were tested. This test system was divided into three parts, exons containing no HPs, HP-containing exons and the 2184 insA mutation

The two assay systems, which were used during the experiments are shown on Fig. 1. To test the analytical performance of the Roche pyrosequencing-based benchtop NGS system (Roche 454 Life Sciences, Branford, CT, USA) in assessing homopolymer sequences, a series of plasmid vectors were generated using pcDNA3.1 as a template (Invitrogen, Life Technologies, Carlsbad, CA). Altogether 12 clones were produced (4-mer, 5-mer, and 6-mer homopolymers of all four nucleotides) using site-directed mutagenesis and following the manufacturer’s instructions (Quikchange II, Agilent Technologies, Santa Clara, CA). In order to avoid the possible generation of length-change mutations during the plasmid replication and the amplification of the specific fragments, PicoMaxx enzyme mix that contains Pfu DNA polymerase (Agilent Technologies, Santa Clara, CA) was utilized. As a quality control step, two colonies of the site-directed mutagenesis were analyzed and confirmed by Sanger sequencing in case of all mutations (Additional file 1: Figure S1).

Each homopolymer tract in the plasmid system was investigated using three pairs of primers (Fig. 1) in order to test the hypothesis, that in the beginning of the sequencing reaction a sufficiently high signal-to-noise ratio might enable precise HP length detection. Our primer design was as follows: i) the homopolymer was located in the vicinity of the forward amplification/sequencing primer in 3′ direction, ii) the analyzed homopolymers were located approximately in the middle of the amplicon and iii) the reverse amplification/sequencing primer’s 3′ end was generated to be as close as possible to the HP segment. Primers used for mutagenesis, amplification, and sequencing are listed in Additional file 2: Table S1. The size of the homopolymer clones varied between 366 and 387 bp, depending on the HP length and the primers used.

In the second set of experiments, a *CFTR* gene mutation detection system was developed and analyzed in detail. 17 clinical samples were used with known *CFTR* mutation status determined by an in vitro diagnostic assay (Elucigene CF29v2, Elucigene Diagnostics, Manchester, UK) and Sanger sequencing. Representative electropherograms of the clinical samples are shown in Additional file 3 Figure S2 and Additional file 4 Figure S3 (all HP-containing exons of the *CFTR* gene and a 2184insA mutation in heterozygous form, respectively, as tested by Sanger sequencing). Primer design for *CFTR* mutation analysis was similar to the plasmid system’s described above, except for the “HP in the middle” type amplicons, which were left out of this experiment (primers listed in Additional file 5: Table S2). Exons e3, e14, e15, and e24 have multiple HP sections, most of which could only be covered within the same amplicons; therefore altogether 33 HP-containing amplicons were tested per patient. When designing the primers, all known single-nucleotide polymorphisms (SNPs) were taken into consideration to maximize annealing efficiency and minimize allele drop-out, which was shown to be an issue in a previous test system [29]. We also designed primers for *CFTR* exons that do not contain homopolymer stretches (Additional file 6: Table S3) to be able to analyze the complete gene. The most crucial section of the gene (within exon 14) was further tested using 11 human DNA samples; including four wild type and seven 2184insA heterozygotes. As with the plasmid system, we used three additional primer pairs to generate amplicons for NGS sequencing with “proximal,” “mid,” and “distal” HP locations. “Proximal” primers were located at a distance of 5 and 11 base pairs from the poly-A tract. In all samples, PicoMaxx enzyme mix was used for the amplification processes.

All human participants gave informed consent for diagnostic genetic analysis. In this study DNA samples were then applied anonymously and procedures were in accordance with the current revision of the Helsinki Declaration. The laboratory is approved by the National Public Health and Medical Officer Service (approval number: 094025024).

Sequencing data of individual reads were evaluated using Amplicon Variant Analyzer software. We differentiated between “proximal”, “mid” and “distal” type of reads referring to HP to sequencing primer distance. Statistics were done using GraphPad Prism v5.03. Numbering of the CFTR exons is based on current recommendations (Ensembl ENSG00000001626). In mutation nomenclature both Human Genome Variation Society (HGVS) and legacy names are used, as suggested [33]. Genotyping accuracy was defined by the percent of correct reads in samples with known genotypes. Acceptable genotyping accuracy was defined to have at least 75% accurate reads of all reads.

## Results

### Quality control of the template preparation

The site-directed mutagenesis experiments were controlled using Sanger sequencing that were performed in duplicates in case of all mutations. No discrepancy was found in any of the introduced mutations (Additional file 1: Figure S1).

### Sequencing homopolymer containing plasmids

**Fig. 2:**
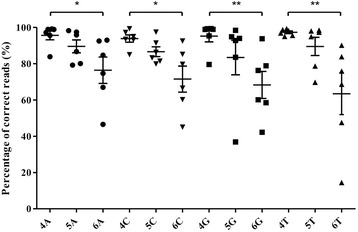
Detection of homopolymers in a plasmid-based system. Read accuracy is expressed as percentage of correct reads. (Kruskal-Wallis test followed by Dunn’s Multiple Comparison Test, * *P* < 0.05 and ** *P* < 0.01)

**Fig. 3:**
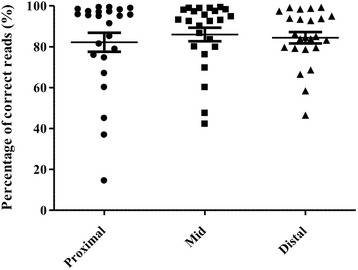
Sequencing primer distance has no effect on homopolymer genotyping. Read accuracy is expressed as average percentage of correct reads. Proximal, equal and distal localization of the homopolymer from the sequencing primer yielded no significant variance in read accuracy. (Kruskal-Wallis test followed by Dunn’s Multiple Comparison Test)

When sequencing homopolymer-containing plasmids, mean coverage was 479 ± 145 and an evident negative correlation was observed between homopolymer length and read accuracy (Fig. 2). The average correct genotyping rate of all four nucleotides combined was 95.8, 87.4 and 72.1% in 4-mers, 5-mers, and 6-mers, respectively (with a 79.6–99.3% range in 4-mers, 36.9–98.4% in 5-mers and 14.5–93.8% in 6-mers). While the pyrosequencing-based NGS system was able to detect poly-A 6-mers reliably (with a mean of 76.4%), detection rates fell under 75% for poly-C, poly-G and poly-T 6-mers (means: 71.5, 68.3 and 63.4%, respectively). In general, longer HPs had lower genotyping accuracy, although, the most accurate reads still reached 98.4% for 5-mers and 93.8% for 6-mers, indicating that careful optimization in a given sequence context might help to skip the poor performing primers and find the most functioning ones for the analysis. After testing, primer localization failed to show any association with genotyping accuracy (Fig. 3).

### Sequencing human DNA samples

Coding regions and exon-intron boundaries in the *CFTR* gene from 6 cystic fibrosis patients with a known mutation status were sequenced. Mean base coverage was 263 ± 178 using our in-house assay. Altogether 246 amplicons were included in the data analysis (> 40 reads).

Although individual read accuracy varied in a wide range (52.2 – 99.1%), average accuracy was generally excellent using the assay (89.3%). The assay was able to detect all small-scale genetic alterations (missense, nonsense, splice site mutations, frameshift/in-frame deletions and insertions) previously identified by Sanger sequencing, providing 100% sensitivity and specificity (Table 1). Regarding the 24 HP stretches the self-designed primer set yielded good performance with more than 80% genotyping accuracy in all but one HP (data not shown). The exception was a 7A HP tract (c.2046_2052) with a 52.2% average correctness.

**Table 1:** Performance of the assay in detecting small-scale alterations in the *CFTR* gene

Location	cDNA position	Legacy name	Mutation class	Sensitivity
i6	c.654-10delAGTT	786-10delAGTT	Splice site	1/1
i6	c.743 + 40A > G	875 + 40A/G	Splice site	1/1
i7	c.869 + 11C > T	1001 + 11C > T	Splice site	1/1
e8	c.926C > G	A309G	Missense	1/1
e11	c.1394C > T	T465I	Missense	1/1
e11	c.1397C > G	S466X	Nonsense	1/1
e11	c.1521_1523delCTT	F508del	In-frame deletion^*^	6/6
e12	c.1624G > T	G542X	Nonsense^*^	1/1
e14	c.2012delT	2143delT	Frameshift deletion	2/2
e14	c.2051_2052delAAinsG	2183AA > G	Frameshift insdel^*^	1/1
e14	c.2052_2053insA	2184insA	Frameshift insertion	6/6
e14	c.2052delA	2184delA	Frameshift deletion^*^	1/1
e15	c.2562 T > G	2694 T/G	Synonymous	4/4
i16	c.2657 + 5G > A	2789 + 5G > A	Splice site^*^	1/1
e17	c.2856G > C	M952I	Missense	1/1
e21	c.3454G > C	D1152H	Missense^*^	1/1
e23	c.3846G > A	W1282X	Nonsense^*^	1/1
e27	c.4389G > A	4521G/A	Synonymous	1/1

Mutations that are included in the Elucigene CF29v2 kit are labelled with an asterisk

**Fig. 4:**
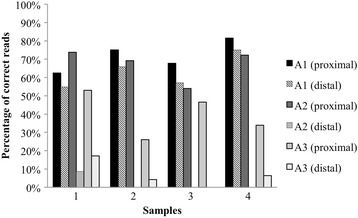
Investigating detection accuracy for 7-mers in exon 14, using three different amplicons for sequencing. The percentage of valid reads in 4 wild type samples is shown from both directions

**Fig. 5:**
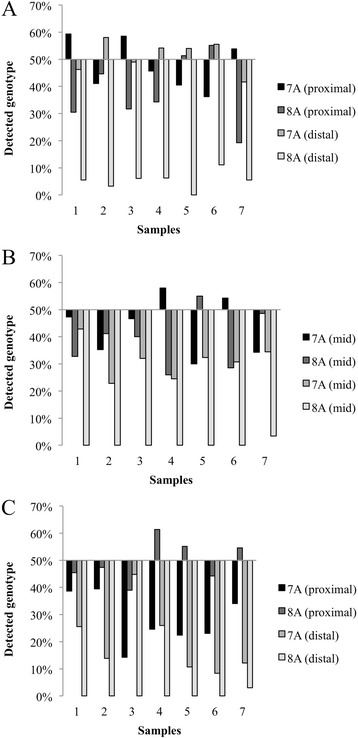
Investigating detection accuracy for 7- and 8-mers in exon 14 using three different amplicons for sequencing. Seven patients heterozygous for 2184insA were analyzed, **a**, **b** and **c** show the degree of deviation of the detected genotypes (7A and 8A) from the theoretical 50%

Therefore a common mutation in this 7A HP (c.2052_2053insA, a 8-mer) was further analyzed using three primer sets (Fig. 1, primer sequences are shown in Additional file 5: Table S2). Depending on the primer used, correct detection of 7A in four wild type DNA samples reached 81%, the percentage of correct reads is shown in Fig. 4. In patients who were heterozygous for the c.2052_2053insA mutation, we calculated the percentage of detected 7A and 8A signals, which, theoretically, should have been 50% for each. The detected genotype frequencies varied in a wide range (16–49%, on average), but the results greatly depended on the primers used for sequence analysis. Two primer pairs (Fig. 5b and c) proved to be rather poor performers with 45–50% irrelevant nucleotide calls. On the other hand, sequencing with a third set of primers, correct 7A and proximal 8A calls were detected with satisfactory accuracy (Fig. 5a), having 27% misreads on average, but even this set could not identify 8 adenines from an approximate distance of 200 bp.

## Discussion

Compared to the Sanger approach, recent next generation sequencing platforms provide deeper coverage and unprecedented throughput. However, inherent drawbacks in terms of read length or false calls could be an issue in some situations [34, 35], which is why careful correction of sequencing errors is crucial [26, 27]. Despite being one of the most common NGS techniques [36], pyrosequencing suffers from over- or undercalls in HP runs, which was described previously [37, 38]. New methods in processing pyrosequencing data might improve the correct analysis of indel mutations even in homopolymer regions [39–41], but it is prudent to be aware of the limitations of medical instrumentation used for investigating patient samples.

In order to assess the accuracy of pyrosequencing in homopolymer regions, first we established a plasmid-based test system. The resulting plasmids contained the most common homopolymers (i.e. 4-mer to 6-mer) as judged by Sanger sequencing. We found that pyrosequencing was more reliable in the case of 4-mers and sequencing of longer HP tracts was increasingly less reliable (see Fig. 2). Reliability unambiguously decreased with the growing number of nucleotides in the homopolymer. Based on our previous experiences, we hypothesized that in the initial phase of the sequencing reaction (immediately following the primers) the signal-to-noise ratio could be acceptably high for the appropriate determination of subtle HP sections. Our results using the plasmid system described was unable to confirm our hypothesis (see Fig. 3). Despite the great variability in sequencing accuracy, we found usable combinations of primers, for all sized homopolymers tested, which highlights the importance of careful primer selection and testing before being used for routine genetic diagnostics.

In the second part we focused on a molecular genetic diagnostic assay development and optimization, where pyrosequencing of the entire coding region of the *CFTR* was carried out. Here we used clinical samples that had been tested using molecular diagnostic-level assays (a CE-IVD kit and Sanger sequencing). The assay showed excellent sensitivity and specificity, because all benign and pathogenic variants (missense, nonsense, splice site alterations, out-of-frame or in-frame deletions and insertions previously identified by Sanger sequencing) were confirmed by pyrosequencing using the in-house designed primer set both in HP and non-HP containing regions. Except for a unique 7A tract, sequencing of 24 HP stretches yielded good performance with genotyping accuracy greater than 80%, including the detection of another 7-mer (7 T) in exon 1 with 83% accuracy. Therefore, it is likely, that homopolymer detection depends not only on HP length or primer distance, but also on several other factors, such as the nucleotide microenvironment in the DNA sequence or the spatial location of beads on PicoTiter plates [42].

It is extremely important to identify even small scale insertions or deletions in homopolymer sections with high reliability. 2184insA, a frequent CF-causing mutation in Hungary and Western Ukraine [30–32] is an eight-adenine long HP region was further analyzed by using 11 DNA samples from CF patients. Other mutations are also known (2184delA and 2183delAAinsG) to affect the same poly-A tract, creating five or six adenine long HPs. We found that mutations that lead to the formation of 5-mers and 6-mers, can be detected with high specificity. While genotyping of the 8-mer c.2052_2053insA is reproducible, the detection rate using pyrosequencing chemistry fails to reach 75% most of the time, therefore this region of the gene still needs to be Sanger sequenced when testing patient samples in routine diagnostic procedures.

## Conclusion

Reliable mutation detection plays a key role when using new NGS techniques for routine clinical diagnostics. In order to reach the required analytical sensitivity and specificity, some NGS methods might need more complex workflow (i.e. the addition of fragment analysis or supplementary Sanger sequencing of certain nucleotide regions). We have developed a plasmid test system that can be used to assess genotyping accuracy of next generation sequencing systems, which could be very useful during the validation of those methods relative to correct homopolymer detection. Through careful planning of PCR primers, we developed an amplicon-based NGS assay that can be used for detection of small-scale *CFTR* mutations and showed, that its analytical validity in the clinical setting is acceptable for 4- to 6-mer HPs, but not for 7-mers and beyond. At the same time, investigation of the 2184insA mutation clearly shows that Sanger sequencing is still required in specific situations and thus, cannot yet be eliminated from the molecular diagnostic workflow.

## Additional files

Additional file 1: **Figure S1.** Representative Sanger electropherograms of the generated homopolymers. 4-mers, 5-mers, and 6-mers are shown in the left, middle, and right columns, respectively. (PDF 176 kb)

Additional file 2: **Table S1.** Mutagenesis, amplification and sequencing primers in the plasmid system. Amplification/sequencing primers contain a starting “Tag sequence,” which was separated by a space within the primer sequence. (DOCX 35 kb)

Additional file 3: **Figure S2.** Representative Sanger electropherograms of all the HP-containing exons of the CFTR gene (clinical sample number 50615). The electropherograms show the exonic sequences including two nucleotides of the introns. (PDF 1945 kb)

Additional file 4: **Figure S3.** Sanger electropherogram of a sample of a patient with a *CFTR* 2184insA mutation in heterozygous form. (PDF 45 kb)

Additional file 5: **Table S2.** Self-designed primers used for assessing HP regions in the CFTR gene. Flopping bases on known SNPs are in brackets. “Tag sequences” also included in the beginning of the primers, separated by a space. (DOCX 58 kb)

Additional file 6: **Table S3.** Self-designed primers used for assessing non- HP regions in the CFTR gene. “Tag sequences” also included in the beginning of the primers, separated by a space. (DOCX 31 kb)

## Abbreviations

CF: Cystic fibrosis
*CFTR*: Cystic fibrosis transmembrane regulator gene
HGVS: Human genome variation society
HP: Homopolymer
NGS: Next generation sequencing
SNP: Single-nucleotide polymorphism
