# Supplementary material for: Analytical parameters and validation of homopolymer detection in a pyrosequencing-based next generation sequencing system
Source: BMC Genomics. 2018 Feb 21;19:158. doi: 10.1186/s12864-018-4544-x (PMC5822529; doi:10.1186/s12864-018-4544-x)

Supplementary figure 1.

Representative Sanger electropherograms of the generated homopolymers

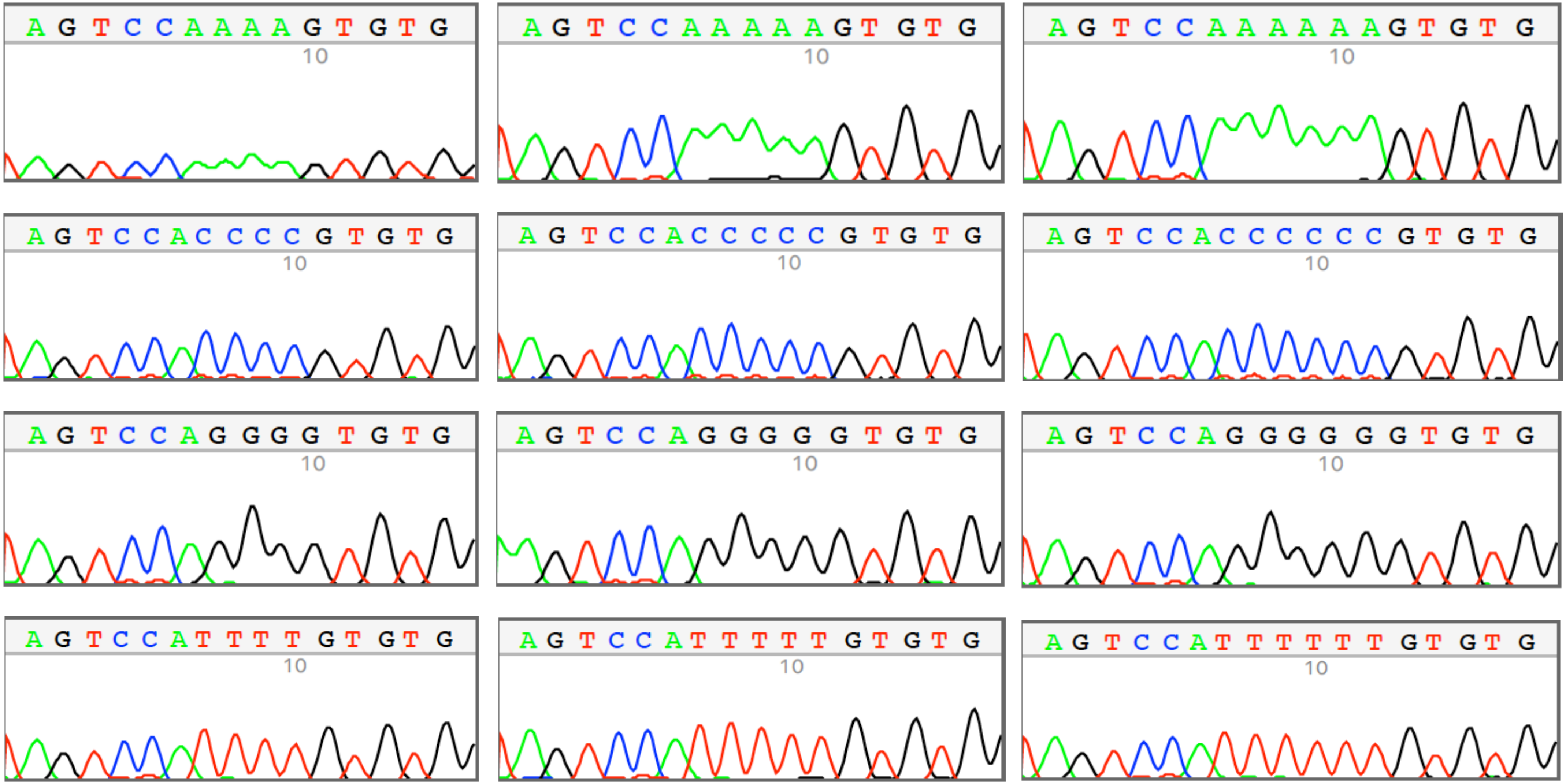

Supplement: Supplementary file 1 — Figure S1. Representative Sanger electropherograms of the generated homopolymers. 4-mers, 5-mers, and 6-mers are shown in the left, middle, and right columns, respectively. (PDF 176 kb) [file 12864_2018_4544_MOESM1_ESM.pdf]
