# Supplementary material for: Analytical parameters and validation of homopolymer detection in a pyrosequencing-based next generation sequencing system
Source: BMC Genomics. 2018 Feb 21;19:158. doi: 10.1186/s12864-018-4544-x (PMC5822529; doi:10.1186/s12864-018-4544-x)
Supplement: Supplementary file 2 — Table S1. Mutagenesis, amplification and sequencing primers in the plasmid system. Amplification/sequencing primers contain a starting “Tag sequence,” which was separated by a space within the primer sequence. (DOCX 35 kb) [file 12864_2018_4544_MOESM2_ESM.docx]

Supplementary Table 1. Mutagenesis, amplification and sequencing primers in the plasmid system. Amplification/sequencing primers contain a starting “Tag sequence,” which was separated by a space within the primer sequence.
