# Supplementary material for: Analytical parameters and validation of homopolymer detection in a pyrosequencing-based next generation sequencing system
Source: BMC Genomics. 2018 Feb 21;19:158. doi: 10.1186/s12864-018-4544-x (PMC5822529; doi:10.1186/s12864-018-4544-x)

Supplementary figure 2.

Representative Sanger electropherograms of all the HP-containing exons of the CFTR gene

50615_CFTR_e1

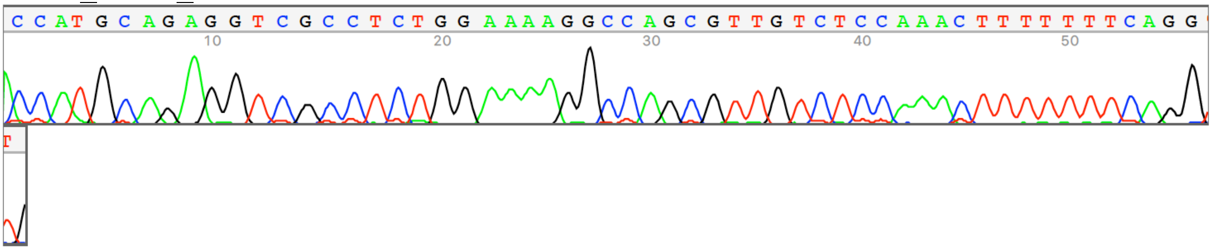

50615_CFTR_e2

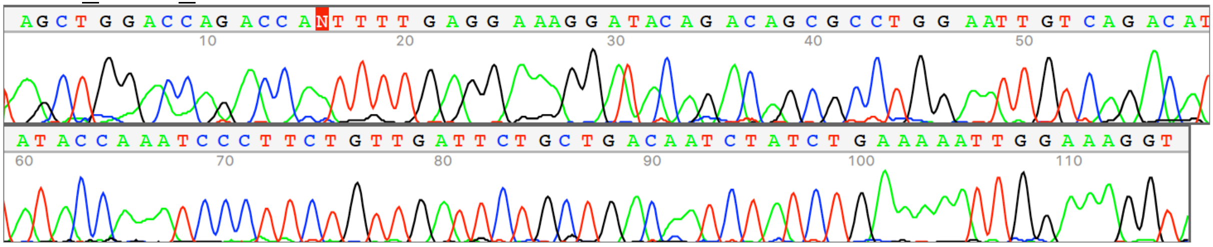

50615_CFTR_e3

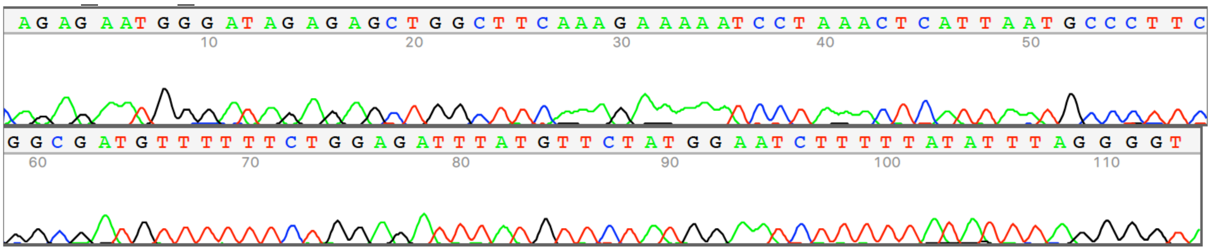

50615_CFTR_e4

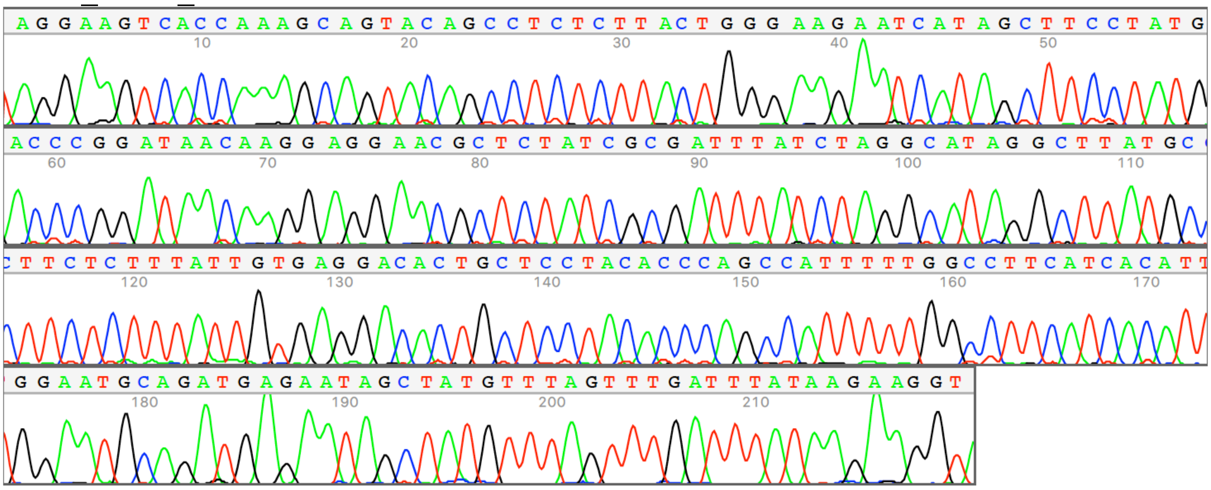

50615_CFTR_e6

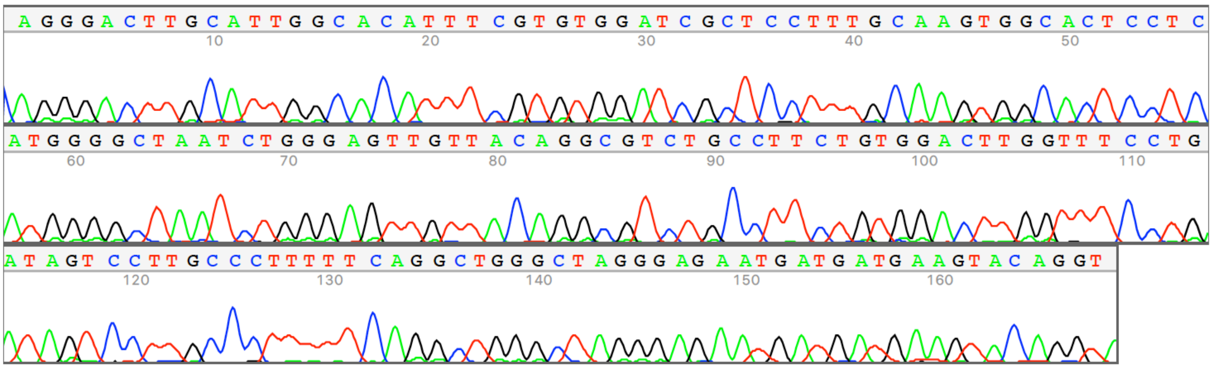


50615_CFTR_e7

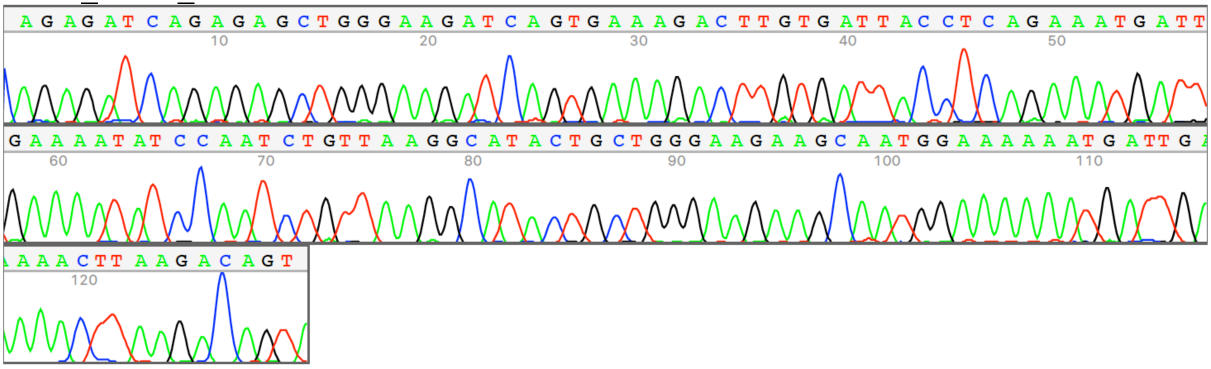


50615_CFTR_e8

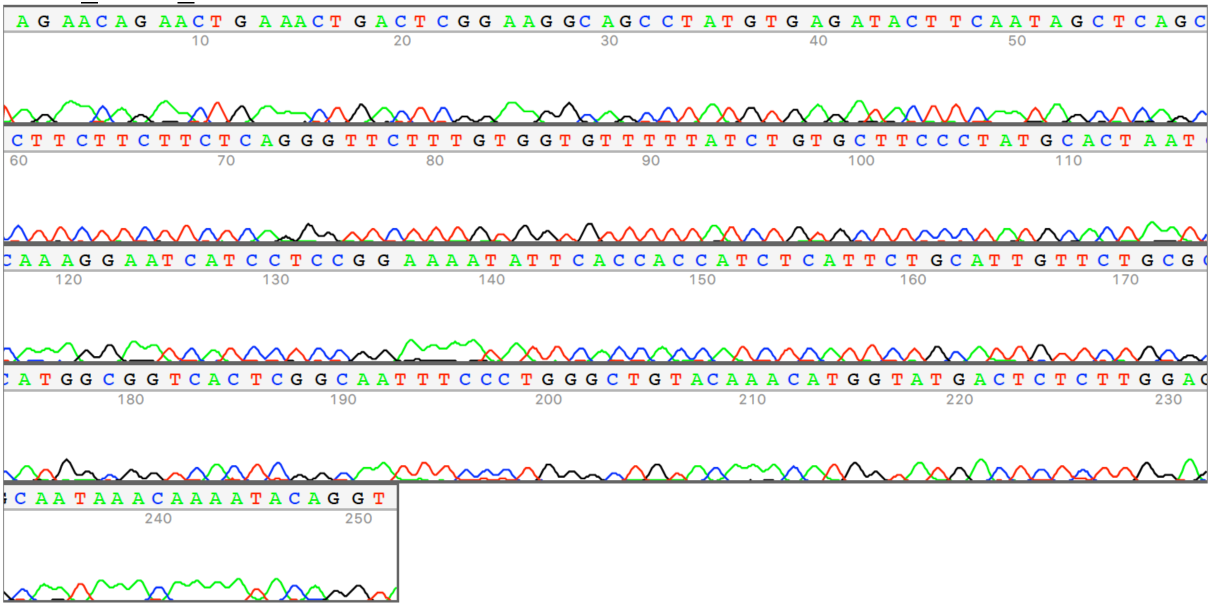


50615_CFTR_e10

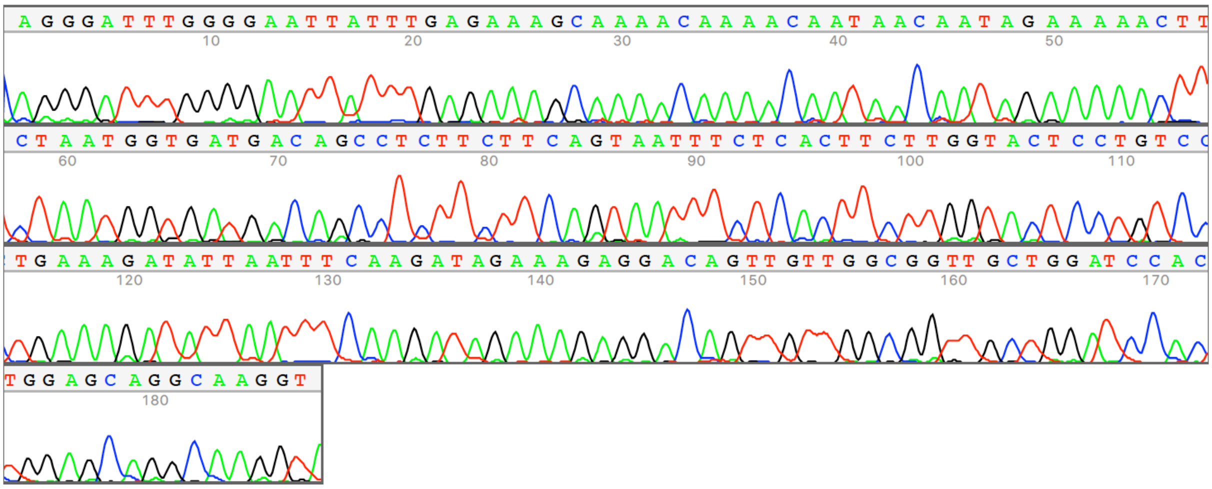

50615_CFTR_e13

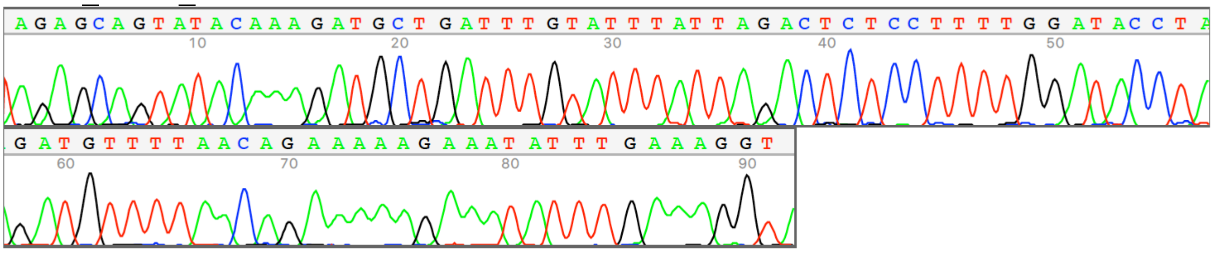

50615_CFTR_e14

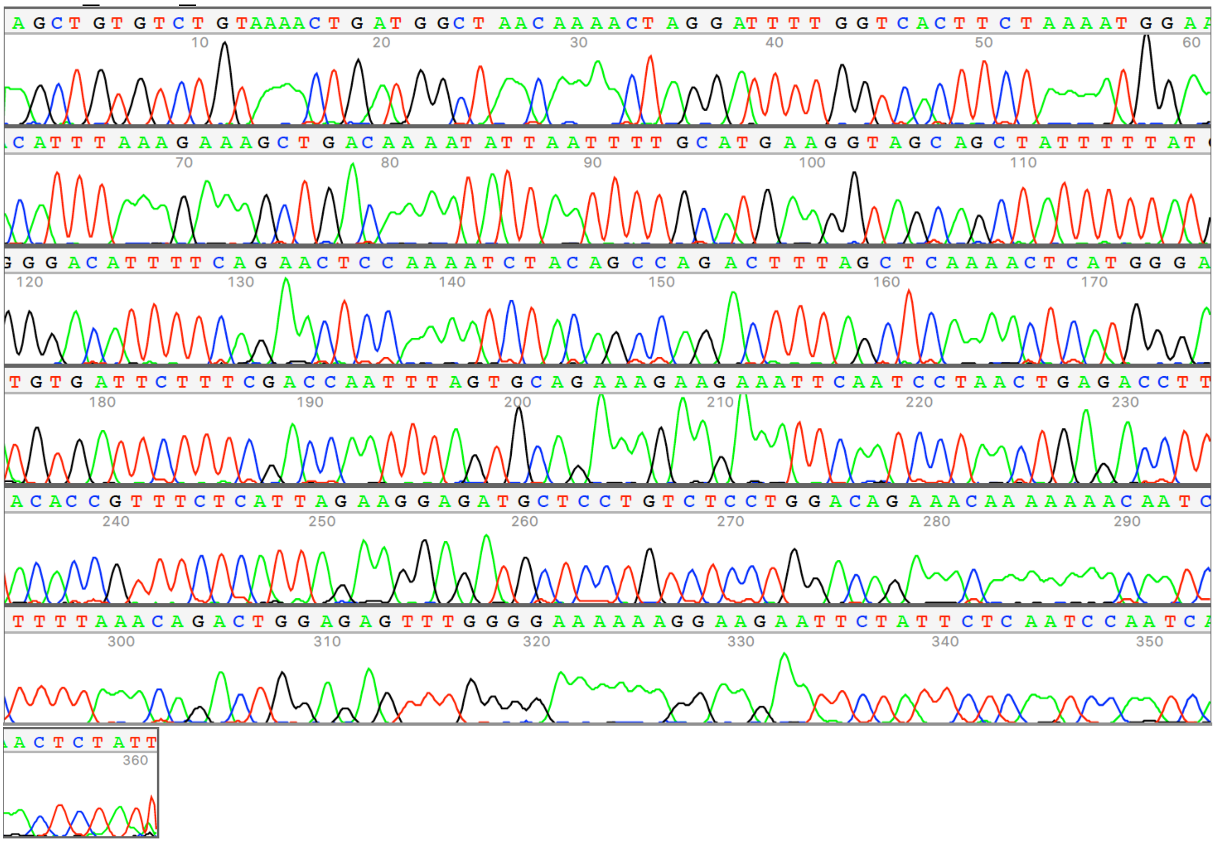

50615_CFTR_e15

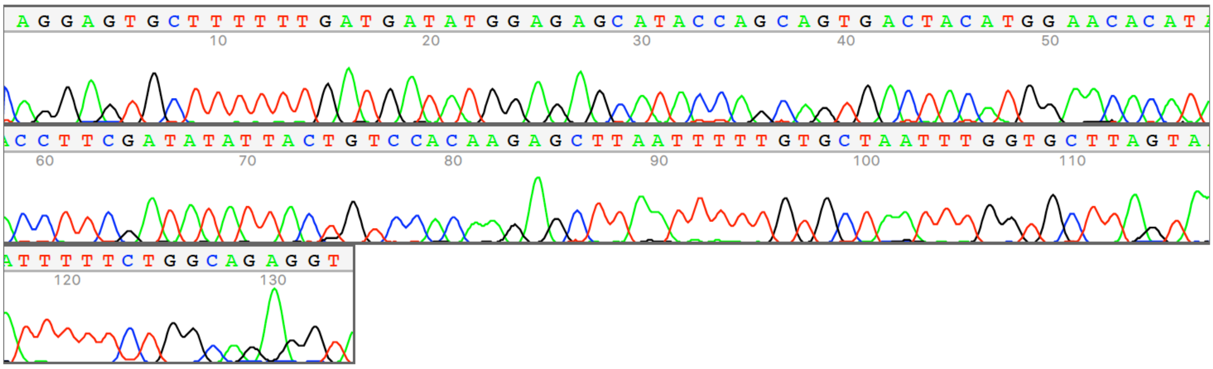

50615_CFTR_e20

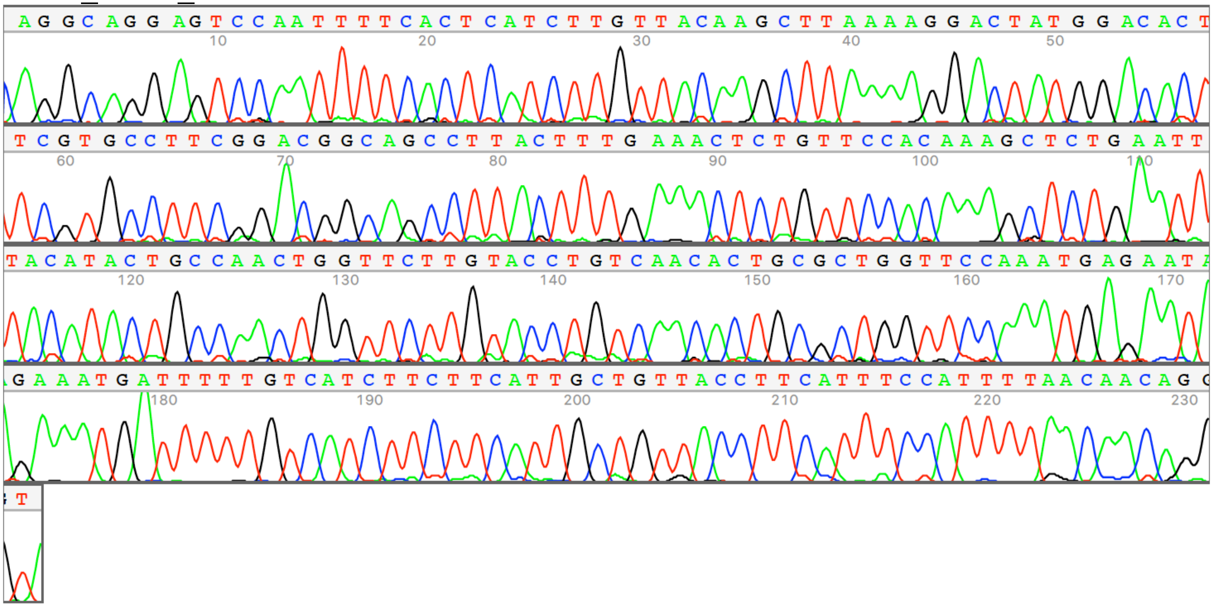

50615_CFTR_e22

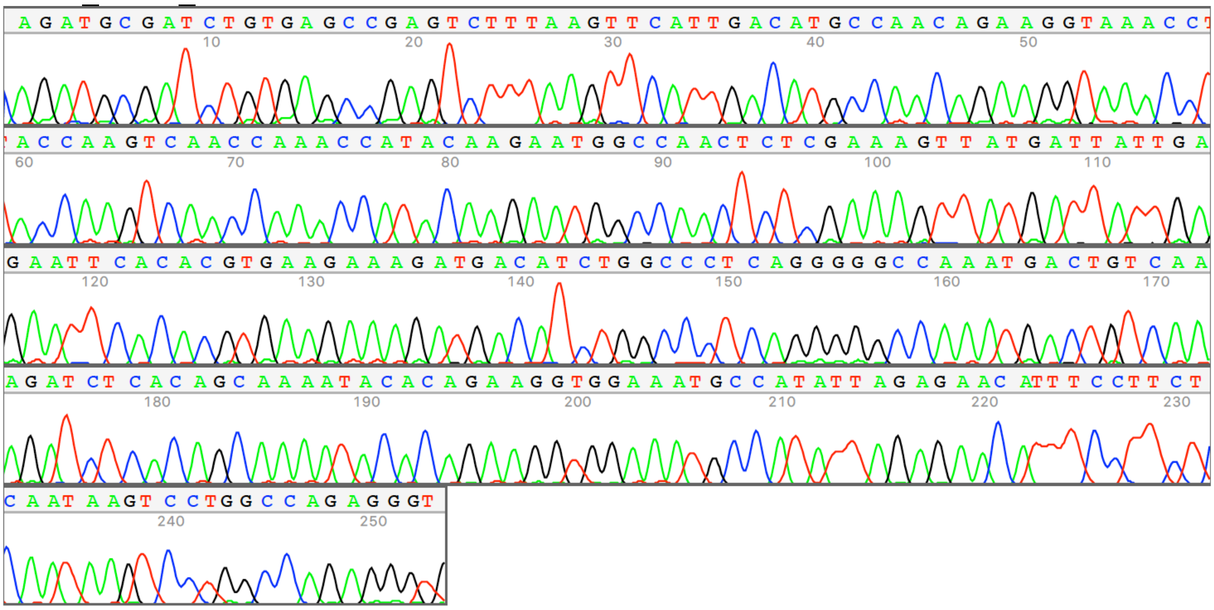

50615_CFTR_e23

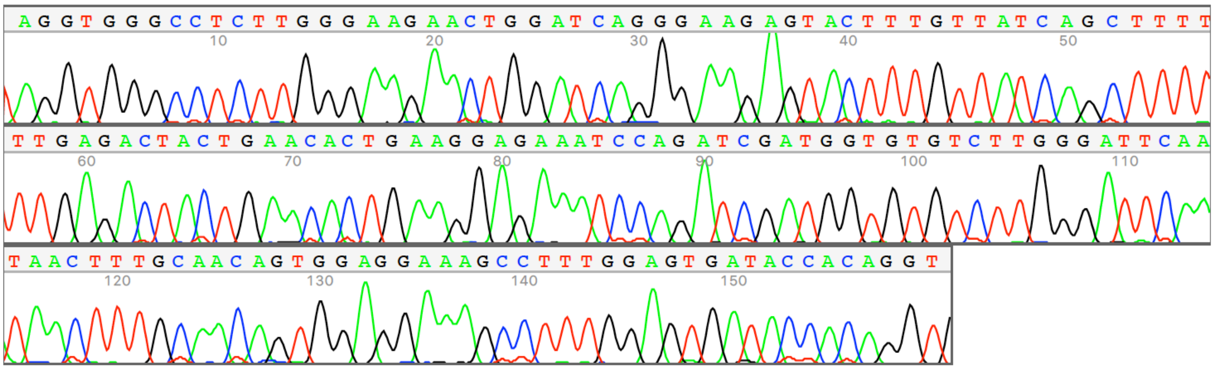

50615_CFTR_e24

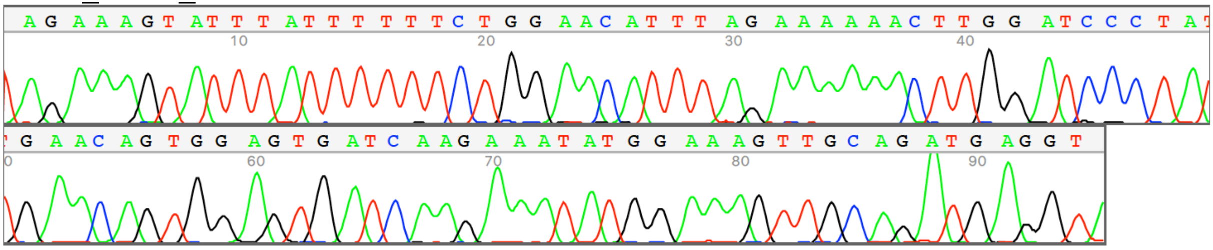

50615_CFTR_e25

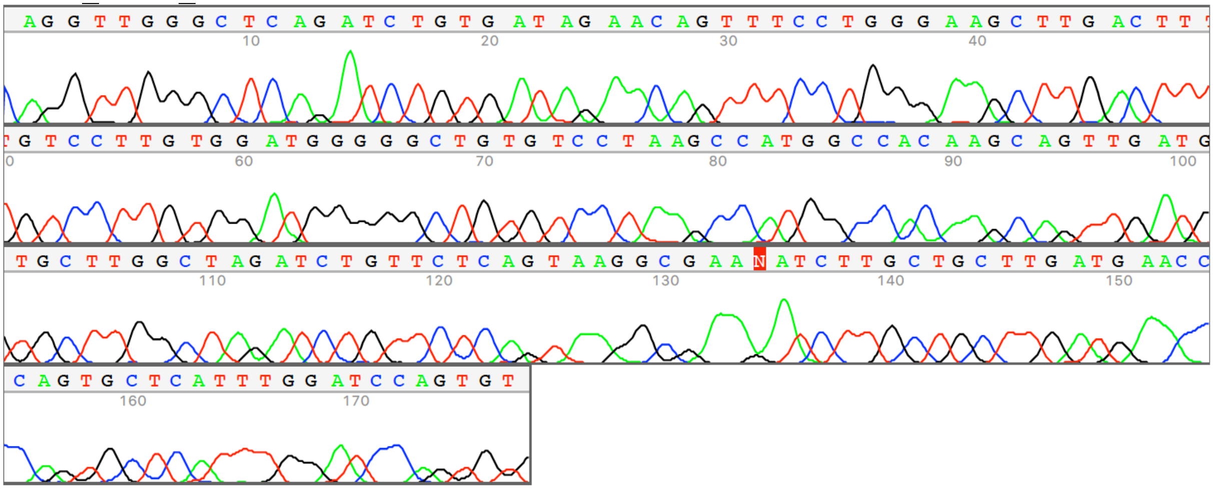

50615_CFTR_e26

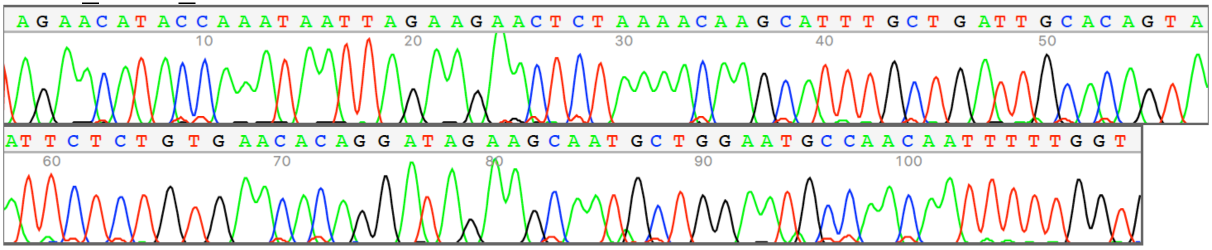

Supplement: Supplementary file 3 — Figure S2. Representative Sanger electropherograms of all the HP-containing exons of the CFTR gene (clinical sample number 50615). The electropherograms show the exonic sequences including two nucleotides of the introns. (PDF 1945 kb) [file 12864_2018_4544_MOESM3_ESM.pdf]
