# Supplementary material for: Analytical parameters and validation of homopolymer detection in a pyrosequencing-based next generation sequencing system
Source: BMC Genomics. 2018 Feb 21;19:158. doi: 10.1186/s12864-018-4544-x (PMC5822529; doi:10.1186/s12864-018-4544-x)

Supplementary figure 3.

Representative Sanger electropherogram of the 2184insA mutation in the CFTR gene

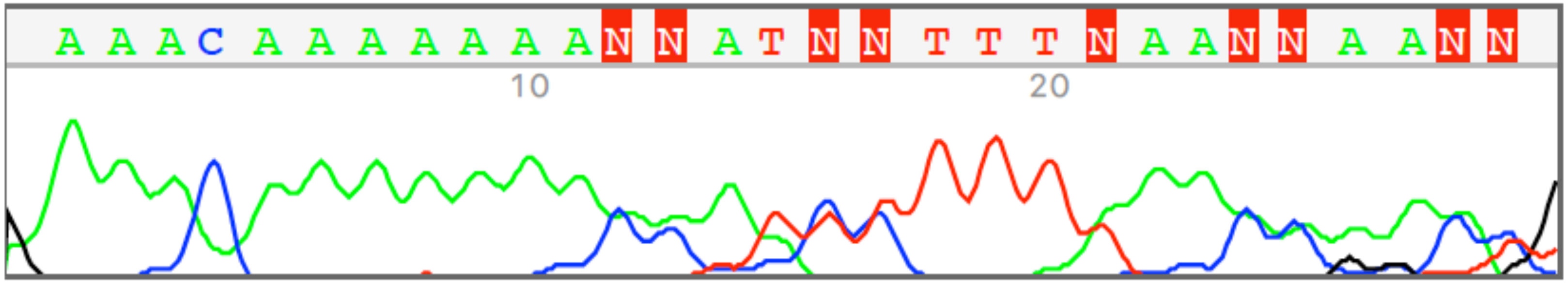

Supplement: Supplementary file 4 — Figure S3. Sanger electropherogram of a sample of a patient with a CFTR 2184insA mutation in heterozygous form. (PDF 45 kb) [file 12864_2018_4544_MOESM4_ESM.pdf]
