# Supplementary material for: Analytical parameters and validation of homopolymer detection in a pyrosequencing-based next generation sequencing system
Source: BMC Genomics. 2018 Feb 21;19:158. doi: 10.1186/s12864-018-4544-x (PMC5822529; doi:10.1186/s12864-018-4544-x)
Supplement: Supplementary file 5 — Table S2. Self-designed primers used for assessing HP regions in the CFTR gene. Flopping bases on known SNPs are in brackets. “Tag sequences” also included in the beginning of the primers, separated by a space. (DOCX 58 kb) [file 12864_2018_4544_MOESM5_ESM.docx]

Supplementary Table 2. Self-designed primers used for assessing HP regions in the CFTR gene. Flopping bases on known SNPs are in brackets. “Tag sequences” also included in the beginning of the primers, separated by a space.
