# Supplementary material for: Analytical parameters and validation of homopolymer detection in a pyrosequencing-based next generation sequencing system
Source: BMC Genomics. 2018 Feb 21;19:158. doi: 10.1186/s12864-018-4544-x (PMC5822529; doi:10.1186/s12864-018-4544-x)
Supplement: Supplementary file 6 — Table S3. Self-designed primers used for assessing non- HP regions in the CFTR gene. “Tag sequences” also included in the beginning of the primers, separated by a space. (DOCX 31 kb) [file 12864_2018_4544_MOESM6_ESM.docx]

Supplementary Table 3. Self-designed primers used for assessing non- HP regions in the *CFTR* gene*.* “Tag sequences” also included in the beginning of the primers, separated by a space.
